# Incidence and Characteristics of Delayed Inflammatory Reactions Secondary to Hyaluronic Acid Filler Injections: A Systematic Review With Meta-Analysis

**DOI:** 10.1093/asjof/ojag165

**Published:** 2026-09-01

**Authors:** Jimyung Seo, Jonathan Kadouch, Tatjana Pavicic, Niamh Corduff, Qin Li, Ada Trindade de Almeida, Clifton Ming Tay, Wei Qi Loh

## Abstract

Delayed inflammatory reactions (DIRs) are rare but increasingly relevant complications of hyaluronic acid (HA) fillers. The aim of this review was to examine the incidence and characteristics of DIRs secondary to HA fillers for facial aesthetic indications. The authors systematically reviewed studies published before December 3, 2024, from PubMed and Embase, using a random-effects meta-analysis model to compute pooled incidence rates of DIR with 95% CIs. Subgroup analyses were performed according to filler crosslinking technology. Case data were analyzed descriptively. The meta-analysis included 242 studies, and case data were supplemented from 71 additional reports. The pooled incidence of DIRs was 0.0005 (95% CI, 0.0002-0.0013) events per patient-years, equivalent to an event occurring for every 2000 patients with 1 year of follow-up. When analyzed by crosslinking technology, incidence rates per patient-year were 0.0044 (95% CI, 0.0006-0.0314) for SHAPE (Stabilized Hyaluronic Acid And Purification Enhancement), 0.0022 (95% CI, 0.0011-0.0043) for Vycross, 0.0007 (95% CI, 0.0003-0.0017) for NASHA (Non-Animal Stabilized Hyaluronic Acid), and 0.0006 (95% CI, 0.0002-0.0025) for HICE (High Concentration Equalized); several filler technologies, including CPM (Cohesive Polydensified Matrix)-based fillers, reported no incident DIR cases during follow-up. The median time to onset was 3 months (range, 2 weeks to 12 years), and time to resolution was 2.9 months (range, 1 day to 6 years). Common presentations included nodules, redness, swelling, and induration. The management often involved hyaluronidase, corticosteroids, and antibiotics. DIRs are rare adverse events following aesthetic HA filler treatments, exhibiting varied times to onset and resolution. Incidence rates appear to vary by crosslinking technology, and awareness of potential differences can contribute to safe and natural outcomes.

**Level of Evidence**: 3 (Therapeutic) 
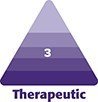

Delayed inflammatory reactions (DIRs) are late-onset hypersensitivity reactions that might develop following the use of hyaluronic acid (HA) filler injectables. Clinically, they present as potentially painful nodules, induration, swelling, and/or erythema that occur ≥2 weeks after filler placement.^1,2^ Although these reactions are perceived as relatively rare, the volume of cases is likely to increase because of the growing global demand for minimally invasive aesthetic procedures, particularly because HA injectables have been utilized not just for volumization but also for skin rejuvenation in recent years, playing a versatile role in aesthetic outcomes.^3-5^

Previous publications have devised treatment algorithms to address DIRs; however, data on their incidence remain limited.^1,2^ A clearer understanding of the epidemiologic landscape of DIRs—in particular, the estimated incidence and clinical characteristics of such reactions—can help guide appropriate management and timely follow-up by practitioners and industry stakeholders. One previous systematic review estimated the incidence rates of DIRs at 1.1 patients per 100 patients-years at risk.^6^ However, the scope of these findings was limited because the analysis was restricted to HA fillers approved by the US FDA, and quantitative methods were not employed. Although DIRs are characterized as infrequent events, quantifying their true incidence has also been challenging because of variations in study designs and follow-up durations.

Moreover, in part because of its rareness, the etiology of DIRs remains poorly characterized. Literature suggests that beyond external immunologic and infectious triggers, product characteristics may influence the risk of delayed reactions.^7^ One review demonstrated that the incidence rates of delayed adverse reactions differ between HA products, possibly influenced by formulation characteristics.^8^ Previous literature has suggested crosslinking technology as one such potential factor.^9^ Efforts to leverage real-world data to further understand product factors that influence DIR onset are critical, considering that fillers are often regulated as medical devices and subject to different pharmacovigilance standards from those of therapeutic drugs.^10,11^

This systematic review and meta-analysis aim to address existing gaps by using quantitative methods to obtain pooled estimates on the incidence of DIRs secondary to aesthetic HA fillers, leveraging data from studies regardless of approval jurisdictions, and analyzing rates according to groupings by crosslinking technology. To complement quantitative findings, we further provide a descriptive clinical picture of DIR by summarizing the case characteristics in literature.

## METHODS

The study protocol was registered in the PROSPERO database (CRD42021260845). We adhered to the PRISMA reporting guidelines for systematic reviews and meta-analyses.

### Search Strategy

A systematic literature search for articles evaluating the safety outcomes of HA filler with a cutoff date up to December 3, 2024, was performed using PubMed and Embase. The search strategy used is described in Supplemental Table 1.

### Eligibility Criteria

We included studies that assessed the safety of HA fillers following the use of aesthetic HA injectables in the facial region in adult patients. For quantitative meta-analysis, prospective and retrospective studies with ≥4 weeks of follow-up that enabled a meaningful estimation of DIR incidence were included. Case reports/series, postmarketing surveillance (PMS) studies, and clinical studies with undefined or <4 weeks of follow-up were retained in descriptive analyses but not the meta-analysis if they reported data on any DIR cases.

Studies were excluded if they (1) assessed HA combined with other therapeutic agents (eg, platelet-rich plasma) rather than the primary intervention, (2) employed alternative HA administration techniques (eg, needle-free jet injectors), (3) did not provide sufficient safety descriptions to assess the number of DIR events (ie, no tabulated number of adverse events [AEs] or explicit statement on DIRs), (4) were based on in vitro or animal data, or (5) were review papers, conference abstracts, or not in English.

### Study Selection and Data Extraction

Two reviewers independently screened titles and abstracts of identified records for relevant results. Discrepancies were resolved by discussion and consultation with a third reviewer. Full-text eligibility was assessed by 2 reviewers, and discrepancies were resolved by discussion.

For each study, the following information were extracted: author's name, publication year, study design, location, number of participants, sex distribution of the population, age, treatment areas, product name, crosslinking technology, follow-up duration, number of DIRs reported, and if any DIR reported: time to onset of event, duration of symptoms, medical interventions taken, and whether or not a biopsy taken. If a biopsy was available, a summary of the histopathological results was extracted. An event was a DIR if it met both of the following criteria: (1) onset time of ≥2 weeks or otherwise specified as “delayed” or “late” by the study and (2) clinical presentation of any of the following: nodules, redness, swelling, induration, erythema, or edema; or was described as inflammatory, hypersensitivity, or an immune-mediated reaction. Additionally, reactions that were diagnosed as a granuloma or foreign body reaction, associated with HA treatment, were also included as DIRs. In order to rule out noninflammatory nodules (eg, product migration), cases with nodules alone that did not present with any other inflammatory symptoms and did not require treatments were excluded.

### Quality Assessment

The Joanna Briggs Institute critical appraisal tools were used to evaluate the risk of bias of individual studies.^12,13^ The risk of bias was considered low when all items were answered “yes,” and high if any item was classified as “no” or “unclear.” Ratings were analyzed descriptively and not used as a criterion for study eligibility.

### Statistical Analysis

The *metarate* function of the *meta* package in R version 4.4.1 was used to perform a meta-analysis of incidence rates. A generalized linear mixed model based on a random-effects model was used to derive pooled estimates. Continuity correction was not applied for studies with zero events considering the rarity of the event being studied. The *I*^2^ statistic was used to evaluate statistical heterogeneity among studies. All other data obtained on the clinical characteristics of DIR events, including case reports, were summarized descriptively.

## RESULTS

### Study Selection

**Figure 1.**
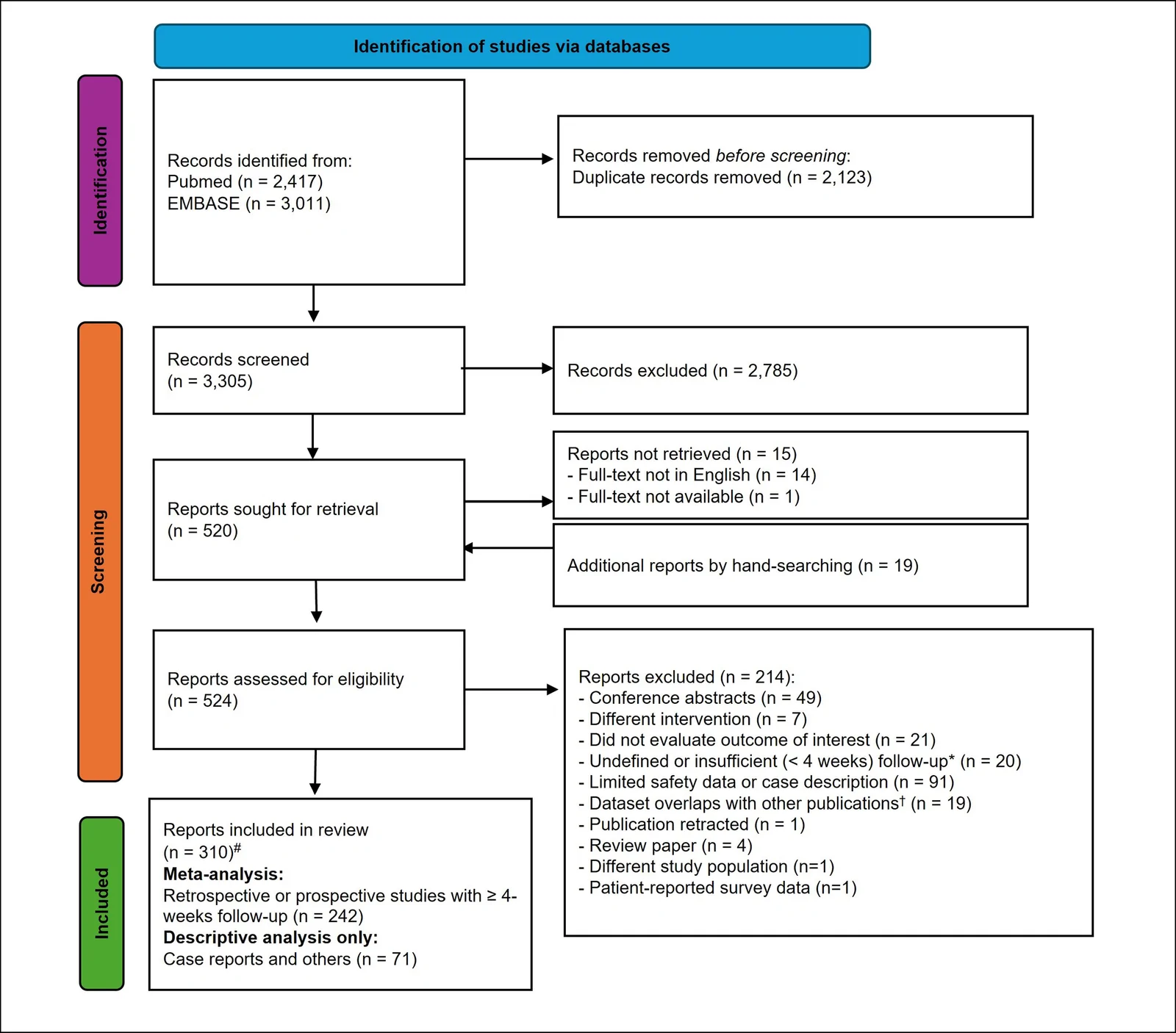
PRISMA flowchart for studies included in this systematic review and meta-analysis. *Studies with undefined or insufficient follow-up that did not report on any definitive cases of delayed inflammatory reaction (DIR). Studies with undefined follow-up but contained individual DIR case data were retained for descriptive analyses. ^†^Where multiple studies reported on the same dataset, the study with the longer follow-up period was retained for analysis. However, data from the shorter-duration study were used for reference and cross-checking. ^#^The total number of studies tabulated from the meta-analysis and descriptive analyses is 3 more than the total number of reports included for review, because 3 reports each contained data from 2 separate prospective studies and were counted as 2 studies each. PRISMA, Preferred Reporting Items for Systematic Reviews and Meta-Analyses.

The initial search yielded a total of 5428 records. After removing duplicates, screening, and full-text review for eligibility, a total of 310 remaining records were retained for data extraction (Figure 1). In total, 242 unique studies were included in the meta-analysis, with 71 additional studies retained for descriptive analyses.

### Meta-Analysis of Incidence

#### Study Characteristics

Characteristics of the studies in the meta-analysis are presented in Table 1 (and detailed in Appendix 1). The majority of the studies were prospective in nature (211 studies [87.2%]), as opposed to retrospective (31 studies [12.9%]). The median duration of the follow-up was 9.9 months (range, 1-60 months), and the median number of patients in a study was 63.5 patients (range, 6-5000 patients). The most common treatment areas were nasolabial folds (93 studies [38.4%]), midface (62 studies [25.6%]), perioral and lower face regions (46 studies [19.0%]), and lips (35 studies [14.5%]). Overall, 30,907 patients were accounted for in these analyses, with a total follow-up of 23,808.55 patient-years.

**Table 1.** Characteristics of Studies Included in Meta-Analysis

Characteristics	Summary statistic
Study design, *n* (%)	
Prospective	211 (87.2)
Retrospective	31 (12.9)
Treatment area,^a^ *n* (%)	
Nasolabial folds	93 (38.4)
Midface	62 (25.6)
Perioral and lower face	46 (19.0)
Lips	35 (14.5)
Upper face	31 (12.8)
Nose	18 (7.4)
Infraorbital hollows/tear trough	19 (7.9)
Atrophic facial scars	6 (2.5)
Geographical region,^a^ *n* (%)	
Europe	91 (36.8)
Northern America	76 (30.8)
Asia	69 (27.9)
Latin America and the Caribbean	7 (2.8)
Oceania	2 (0.8)
Africa	2 (0.8)
Median duration of follow-up, in months (range)	9.9 (1, 60)
Median number of patients (range)	63.5 (6, 5000)
Total number of patients	30,907
Total number of patient-years follow-up	23,808.55

^a^Characteristics comprising nonmutually exclusive categories.

#### Estimated Incidence Rates

**Figure 2.**
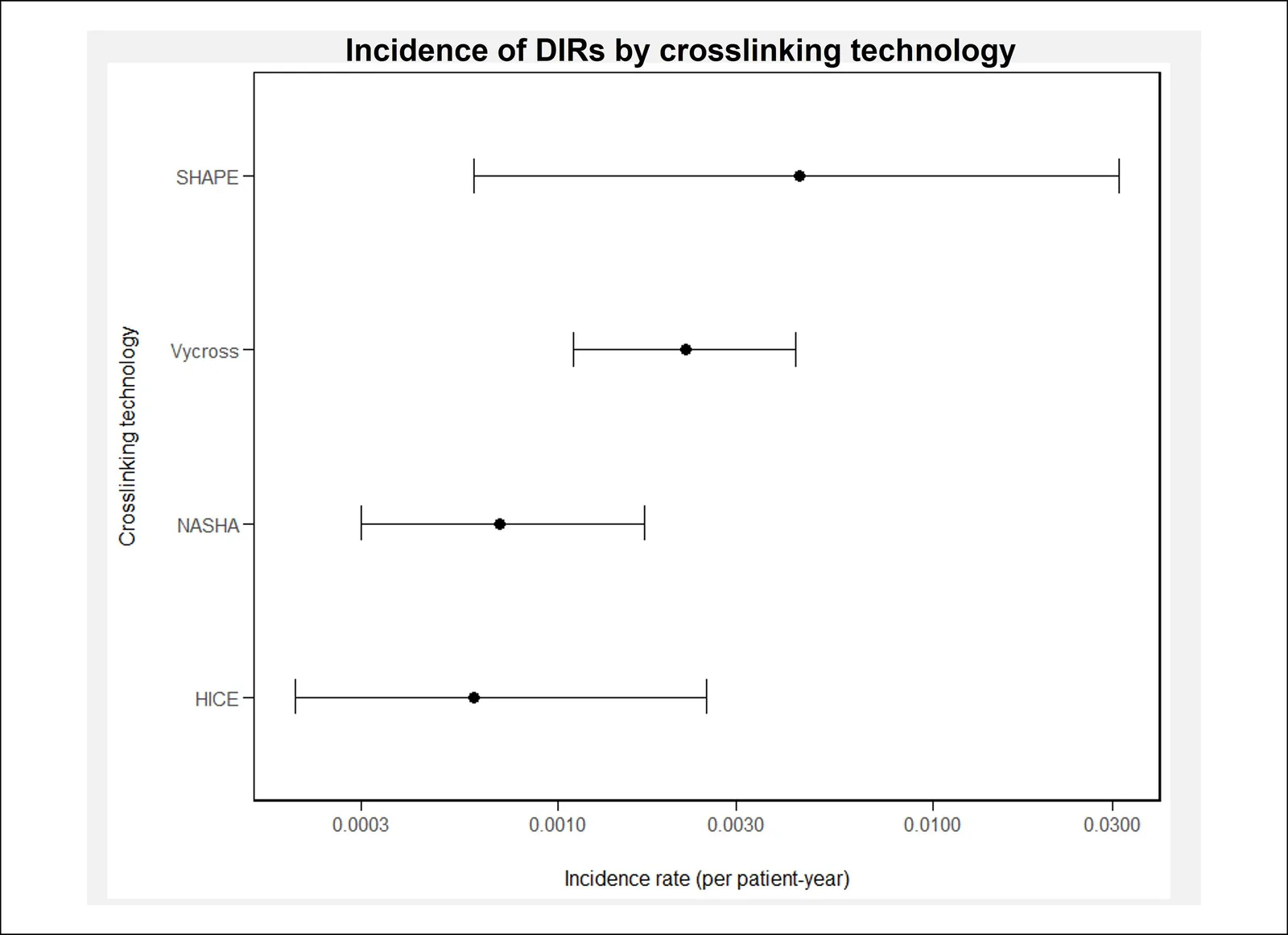
Visual representation of incidence of delayed inflammatory reactions (DIRs) by crosslinking technology. Points represent estimated incidence rates per patient-year and horizontal lines indicate 95% CIs. Only crosslinking subgroups with at least 3 included studies are depicted, and subgroups reporting zero events were excluded. CI, confidence intervals; HICE, High Concentration Equalized; SHAPE, Stabilized Hyaluronic Acid and Purification Enhancement; NASHA, Non-Animal Stabilized Hyaluronic Acid.

A total of 32 cases of DIR were reported over follow-up across the 242 studies. Pooled estimates of the incidence rates of DIR were 0.0005 events per patient-year (95% CI, 0.0002-0.0013) with an *I*^2^ of 0.0% (95% CI, 0.0%-16.4%); equivalent to an event occurring for every 2000 patients with 1-year follow-up. Varying incidence rates were observed in subgroup analyses based on crosslinking technologies (Table 2, Figure 2). When ordered by pooled point estimates, the DIR incidence computed for respective HA filler technologies were 0.0044 events per patient-year (95% CI, 0.0006-0.0314) for SHAPE (Stabilized Hyaluronic Acid And Purification Enhancement) technology, 0.0022 events per patient-year (95% CI, 0.0011-0.0043) for Vycross technology, 0.0007 events per patient-year (95% CI, 0.0003-0.0017) for NASHA (Non-Animal Stabilized Hyaluronic Acid) technology, and 0.0006 events per patient-year (95% CI, 0.0002-0.0025) for HICE (High Concentration Equalized) technology. Several crosslinking technologies reported no incident DIR cases during follow-up, including Hylacross technology, as well as CPM (Cohesive Polydensified Matrix)-based fillers, which also had the largest number of contributing studies (*n* = 31).

**Table 2.** Incidence Rates of DIR in Subgroups According to Crosslinking Technology, Sorted in Descending Order by Number of Studies

Group	No. of studies	Total patient-years	No. of events	Rate (per patient-year)	95% CI	Heterogeneity
NASHA	93	6951.6	5	0.0007	0.0003-0.0017	*I* ^2^ 0.0% (0.0%-25.2%); *Τ*^2^ = 0
Vycross	53	6493.2	14	0.0022	0.0011-0.0043	*I* ^2^ 0.0% (0.0%-32.2%); *Τ*^2^ = 0.0008
CPM	31	1806.7	0	—	—	—
OBT	25	1563.0	0	—	—	—
Hylacross	12	1057.9	0	—	—	—
HICE	10	3168.6	2	0.0006	0.0002-0.0025	*I* ^2^ 0.0% (0.0%-62.4%); *Τ*^2^ = 0
SMART	8	656.5	0	—	—	—
PNT	6	731.4	0	—	—	—
NAHYCO	4	63.8	0	—	—	—
SHAPE	3	226.0	1	0.0044	0.0006-0.0314	*I* ^2^ 0.0% (0.0%-89.6%); *Τ*^2^ = 0
Tri-Hyal	3	285.5	0	—	—	—
4L	3	220.0	0	—	—	—

Only crosslinking technology subgroups with ≥3 clinical studies were included for presentation in this table.

CPM, cohesive polydensified matrix; DIR, Delayed Inflammatory Reactions; HICE, High Concentration Equalized; NASHA, Non-Animal Stabilized Hyaluronic Acid; OBT, Optimal Balance Technology; PNT, Preserved Network Technology; SHAPE, Stabilized Hyaluronic Acid and Purification Enhancement; SMART, Supreme Monophasic and Reticular Technology (Note: also referred to as the MAtrix CROsslinking Core Technology).

### Descriptive Analyses

#### Clinical Characteristics of Delayed Inflammatory Reactions Cases

In total, information on 238 individual cases was extracted (Appendix 2). The majority of the cases were females (97.5%), and the median age was 48.0 years (range, 22.0-77.0 years). The time to onset of DIR varied greatly, with a median of 3 months (range, 2 weeks to 12 years). The most frequently reported symptoms were nodules (often described as palpable), swelling, redness, and induration. Accompanying tenderness or pain was reported in some cases.

**Figure 3.**
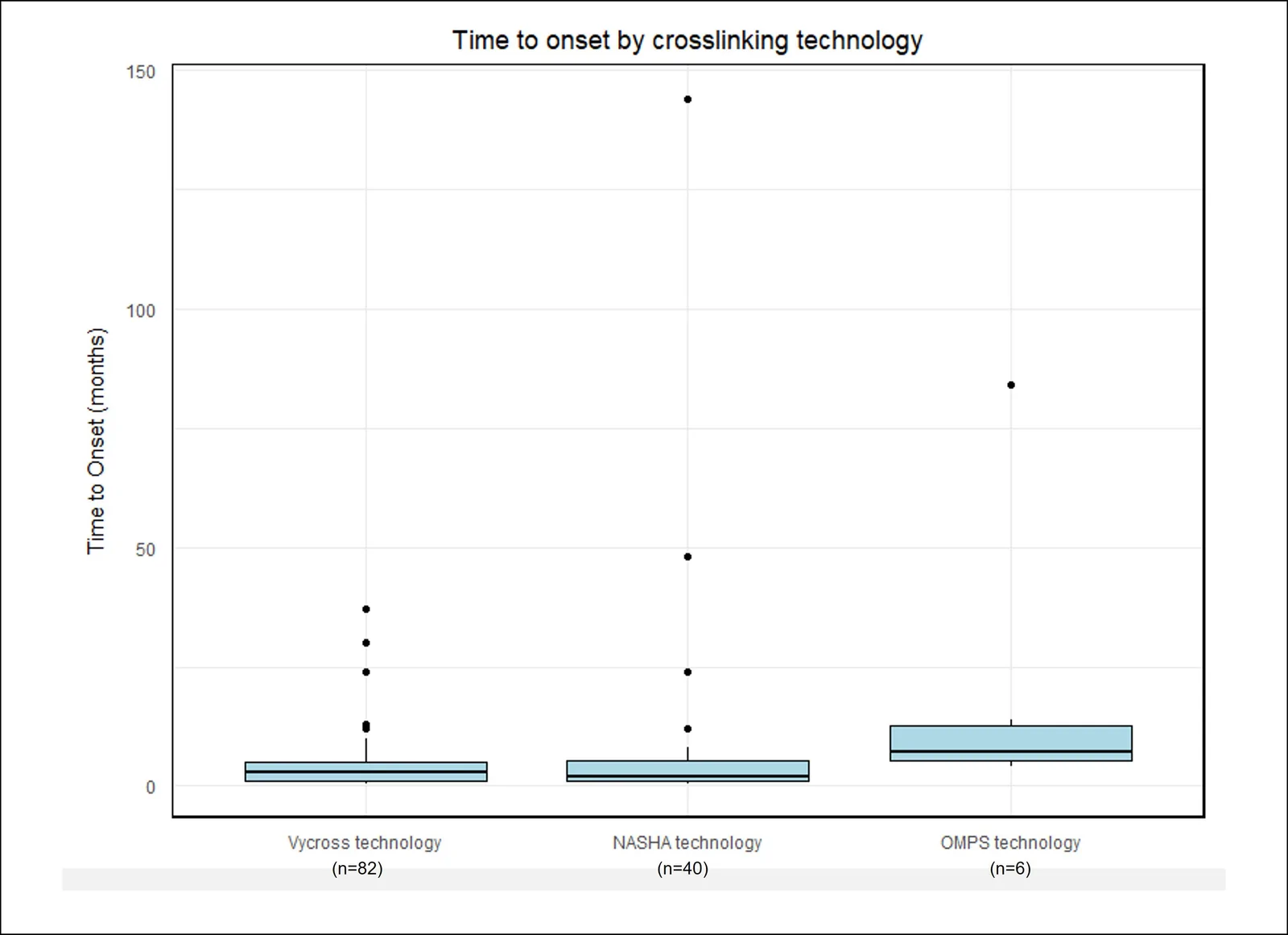
Boxplot diagram on the distribution of time to onset (months) of delayed inflammatory reactions (DIRs) by crosslinking technology for Vycross (*n* = 82), Non-Animal Stabilized Hyaluronic Acid (NASHA; *n* = 40), and OMPS (*n* = 6). Other crosslinking technologies were excluded in this visual representation because of insufficient data (≤5 cases of DIR in total) precluding meaningful statistical summary. Points represent outliers, defined as values more than 1.5 of the interquartile range from the box hinges.

When grouped by crosslinking technologies, the largest number of DIRs were reported following treatment with HA fillers manufactured based on Vycross (*n* = 82), NASHA (*n* = 40), and PureSense (also referred to as OMPS; *n* = 6) technologies, with the distribution of time to onset shown in Figure 3. Notably, studies reported differential local reactions within an individual when fillers of different crosslinking technologies were administered in the same session.^14,15^ In 1 case, all areas treated with an HA filler crosslinked with PureSense technology showed diffuse swelling of an inflammatory nature, whereas the lips, treated with a CPM-based HA filler, remained unaffected.^14^ Another individual developed firm swelling in areas treated with a Vycross-based HA filler, with no cross-reaction to a Hylacross-based product administered on the same day; these differential reactions were reproduced subsequently in intradermal testing in the forearm.^15^

Frequently reported management strategies included hyaluronidase, corticosteroids (most often administered systemically, although intralesional injections were also used), antibiotics, and surgical excision. Antihistamines, nonsteroidal anti-inflammatory drugs, antineoplastic agents (eg, 5-fluorouracil), and aspiration were sometimes employed. For several cases, ultrasound imaging was used to guide the intralesional delivery of hyaluronidase or corticosteroids. Although complications with treatments were rare, there was one case of suspected iatrogenic Cushing's disease, presenting with muscle weakness and fatigue as well as bilateral neck masses following the prolonged use of prednisolone for the management of DIR.^16^ The time to resolution of symptoms varied greatly, with a median of 2.9 months (range, 1 day to 6 years). Although some cases were transient and resolved shortly with treatment, others were characterized by recurrent symptoms that further exacerbated over time, requiring long-term treatment and, in some cases, surgical excision. In a case series, patients initially diagnosed with DIR following aesthetic HA injections went on to develop systemic responses resistant to treatments characterized as autoimmune/inflammatory syndrome induced by adjuvants (ASIA) syndrome.^17^ A predisposition to autoimmunity, or previous adjuvant exposure, was identified as a potential risk factor for such severe late-onset systemic responses.

Histological findings were available for slightly less than a quarter of all cases (22.3%). Most of these findings demonstrated granulomatous inflammation or characteristics of a foreign body reaction. Generally, histological observations comprised an inflammation infiltrate marked by the involvement of multinucleated giant cells, macrophages, lymphocytes, neutrophils, and/or histocytes. In several cases, amorphous or acellular basophilic deposits of foreign material identified as HA were present, and surrounding areas of fibrosis could be observed. Eosinophilia was also reported in several case descriptions.

#### Aggregate, Exposure-Level Frequency Data

Six studies presented data on the cumulative risk of DIR (Table 3). Three studies leveraged AE reports from PMS databases and/or literature reviews, 1 study employed practitioner surveys, and 2 studies comprised large-scale retrospective reviews of medical records. Reported frequencies varied in denominators (eg, syringes and AE reports) and outcome type (eg, granulomas or inflammatory nodules).

**Table 3.** Studies Providing Summaries of Cumulative Risk of DIR

Author	Study title	Product	Crosslinking technology	Data source	Exposure	No. of DIR events (clinical presentations)	Histological data	Interpretable findings
Jones et al (2020)^18^	Five-year postmarket safety experience with the Optimal Balance Technology range of hyaluronic acid fillers	Restylane Refyne, Restylane Kysse, Restylane VOlyme, and Restylane Fynesse	OBT	Spontaneous AE reporting during PMS, in combination with AEs reported in literature	922,594 syringes; 5-year period (January 2011 to December 2015)	28 delayed-onset AE (nodules, granuloma, mass, induration, and swelling/inflammation with/without infection)	3 out of 28 events histologically confirmed granulomas	AE frequency per syringe: 1 delayed-onset AE per ∼33,000 syringes
André (2004)^19^	Evaluation of the safety of a non-animal stabilized hyaluronic acid (NASHA—Q-Medical, Sweden) in European countries: a retrospective study from 1997 to 2001	Restylane	NASHA	Practitioner surveys	12,344 syringes; 4-year period (from 1997 to 2001)	18 delayed hypersensitivity reactions (redness with inflammatory/noninflammatory nodules, sometimes presenting with an abscess)	—	AE frequency per syringe: 1 delayed reaction per ∼686 syringes
Michel et al (2023)^20^	Are delayed dermal filler granulomas more common since COVID-19?	Various fillers including Juvederm (Voluma, Vollure, Volbella, Ultra, and Ultra Plus), and Restylane (L, Kysse and Lyft)	Vycross technology, Hylacross, NASHA, or OBT	Retrospective review of medical records from practice	3525 patients and 8067 syringes; 3.2-year period; from August 1, 2018 to October 31, 2021	11 events (7 with Vollure, 4 with Voluma) identified as likely granulomas in 9 patients; onset of >2 weeks	—	AE frequency per syringe: 1 delayed granuloma in ∼730 syringes, AE frequency per patient: 1 delayed granuloma in ∼320 patients
Kern et al (2022)^21^	Serious adverse events with injectable fillers: retrospective analysis of 7659 patient outcomes	Not specified	Not specified	Retrospective analysis of serious AEs from multipractitioner outpatient clinic	Estimated 15,000 HA syringes; 11.58-year period (from January 2009 to August 2020)	3 serious delayed AEs (time to onset was average of 5.5 months from recent injection; delayed nodules); all following use of filler based on Vycross technology: 2 Juvederm Voluma, 1 Juvederm Volbella	—	AE frequency per syringe: 1 serious delayed AE per ∼5000 syringes
Siperstein et al (2024)^22^	Long-term 23-year global post-marketing safety surveillance review of delayed complications with a supportive hyaluronic acid filler for infraorbital hollow rejuvenation	Restylane	NASHA	Global manufacturer PMS database	7972 reports of AEs; 23-year period (from 1999 to 2022)	134 hypersensitivity reactions, 46 inflammatory nodules, 10 granulomas (time to onset ranged from 15 days to 3 years, with median of 2 months)	—	Proportion of AE: DIRs make up ∼2.4% of all AEs reported
Cohen et al (2022)^23^	Postmarket safety surveillance of delayed complications for recent FDA-approved hyaluronic acid dermal fillers	Restylane, Restylane Refyne, Restylane Defyne, Juvéderm Vollure, Juvéderm Volbella, Teosyal RHA 2, Teosyal RHA 3, Teosyal RHA 4, Restylane Kysse, Revanesse Versa	OBT, NASHA, Vycross technology, PNT, Thixofix technology	MAUDE manufacturer PMS database	A combined total of 585 AE reports	OBT: 2 granulomas, 5 hypersensitivities, 6 inflammatory nodules Vycross technology: 10 granulomas, 34 hypersensitivities, 69 inflammatory nodules Thixofix technology: 0 events PNT: 1 granuloma, 3 hypersensitivity, 7 inflammatory nodules	—	Proportion of AE: Vycross technology made up majority of events reported; 10/13 (76.9%) of granulomas, 34/42 (81.0%) of hypersensitivities and 69/82 (84.1%) of inflammatory nodules

AE, adverse event; COVID-19, coronavirus disease 2019; DIR, delayed inflammatory reactions; FDA, Food and Drug Administration; HA, hyaluronic acid; NASHA, Non-animal stabilized hyaluronic acid; MAUDE, manufacturer and user facility device experience; OBT, Optimal Balance Technology; PMS, postmarketing surveillance; PNT, Preserved Network Technology; RHA, Resilient Hyaluronic Acid.

Among studies reporting event frequency based on syringes, Jones et al reported 28 delayed-onset AEs across an estimated 922,594 syringes (∼1 event per 33,000 syringes) for HA fillers manufactured with Optimal Balance Technology.^18^ In contrast, André reported a higher cumulative risk of 18 delayed reactions across 12,344 syringes (∼1 event per 686 syringes) for NASHA-based HA fillers.^19^ A study in an outpatient clinic reported 11 cases of delayed granulomas following the administration of 8067 syringes of HA fillers based on multiple technologies (∼1 event per 730 syringes); notably, all events had occurred following use of a Vycross-based HA filler.^20^ In a retrospective analysis specific to serious delayed AEs,^1^ 3 events, all subsequent to the use of a Vycross-based filler, were identified following administration of an estimated 15,000 syringes of HA fillers of varying crosslinking technologies (∼1 event per 5000 syringes).^21^

When evaluating cumulative AE reports, events relating to DIRs (delayed hypersensitivities, inflammatory nodules, and granulomas) represented ∼2.4% of all AE reports (*n* = 7972) in 1 PMS study for Restylane crosslinked with NASHA.^22^ In an analysis of PMS data for 5 FDA-approved crosslinking technologies, fillers based on Vycross technology accounted for the majority of the events: 10 of 13 granulomas (76.9%), 34 of 42 hypersensitivity cases (81.0%), and 69 of 82 cases of inflammatory nodules (84.1%); however, as data on the frequency of use was not available, limited conclusions could be made regarding true incidence.^23^

## DISCUSSION

Because the demand for minimally invasive cosmetic treatments continues to rise, efforts to examine risk profiles and monitor trends over time are increasingly important. Aesthetic procedures are typically elective interventions as opposed to therapeutic treatments, and under such circumstances, the principle of nonmaleficence is most pertinent and underscores the need for thorough evaluations of safety. Among the spectrum of AEs observed with HA treatments, DIRs represent a group of unpredictable and late-onset complications, which are challenging to treat and can lead to long-term detriments on quality of life.^24^ Notably, the risk of DIR has been frequently associated with product factors, as opposed to other complications—such as vascular occlusion—which arise from procedural risks.^25^ Therefore, our study aimed to derive a comprehensive estimate of the incidence and characteristics of these events, including product-specific evaluations, to help inform risk–benefit assessments.

In this study, our pooled estimate of overall DIR incidence was 0.0005 (95% CI, 0.0002-0.0013) events per patient-years, equivalent to an event occurring for every 2000 patients with 1-year follow-up, indicating that DIRs are a relatively rare event. Our estimates are less than those derived from a previous review, which may partly be because of the use of a narrower definition of DIR.^6^ Our study focused on events occurring ≥2 weeks after filler implantation and with specific clinical presentations of inflammation, whereas previous work had employed a broader definition of delayed reactions.

Importantly, our findings indicated variability in incidence according to filler crosslinking technologies. This was consistently observed in the pooled incidence rates from the quantitative meta-analysis and the number of incident cases extracted. For some crosslinking technologies, the absence of reported events may reflect limited clinical investigation rather than a lower incidence. However, variability in incidence persisted even among the most frequently studied filler technologies, each supported by an extensive body of clinical studies.

Earlier evidence suggests that crosslinking technologies using a higher proportion of low molecular weight (LMW) HA (molecular weight <1000 kDa) as starting ingredients may be more prone to immune-mediated inflammatory reactions.^26-29^ LMW-HA fragments from long-term breakdown of filler products may play a role in the development of DIR because of the activation of pro-inflammatory pathways.^26,30-32^ Our results are aligned with these hypotheses, because the highest number of incident cases were reported for HA fillers based on Vycross technology, which employs a majority of LMW-HA as a starting material. The pooled estimated rates for this crosslinking technology were equivalent to about 1 case for every 450 patients with 1 year of follow-up. Although informative, these findings should still be interpreted with caution because the rarity of these events limits the precision of quantitative estimates.

Beyond the composition of LMW-HA, the heterogeneity in our data points toward other product-specific factors that influence incidence rates. Studies suggest that impurities present in the manufacturing processes, or even residual endotoxin, may influence a product's propensity toward DIRs.^7^ Notably, Lee et al previously detected concerning levels of silicon, aluminum, and iron impurities, which have been associated with immunological reactions, in several commercially available HA fillers.^33^ More recently, an integrated study of HA fillers based on CPM technology demonstrated low levels of insoluble particles beneath that of pharmacopeial thresholds and exhibited favorable tissue integration with an absence of inflammatory reactions in histological assessments. These findings are consistent with the low incidence of DIRs with CPM technology reported in our systematic review and meta-analysis.^34^

Based on consolidated case descriptions, the onset and course of a DIR can vary greatly between individuals. This emphasizes that DIRs comprise a heterogeneous group of inflammatory responses rather than a uniform clinical entity and that an individualized approach to diagnosis and management and long-term monitoring may be appropriate rather than a standard treatment algorithm.

Previously described treatment algorithms share a common underlying framework of initial antibiotic treatments, followed by potential escalation to hyaluronidase and intralesional therapies, but differ in treatment sequencing, use of combination treatments, and the role of steroids.^1,2,35^ In practice, management often involves iterative adjustment of therapies based on clinical response and integrating further investigations (eg, ultrasound and cultures) to guide diagnosis and treatment.^36^ Our findings suggest that awareness of the injected filler's crosslinking technology may further assist clinicians in weighing the likelihood of an infectious vs a filler-related immune-mediated etiology, thereby helping to guide the choice and sequencing of treatment.

One key limitation of our analysis is that DIR incidence over follow-up is likely to be underreported within the current literature. Given that substantial time may elapse before onset and that the typical follow-up duration of a clinical study may not be able to capture events, it remains challenging to derive an accurate estimate of DIR incidence.^37,38^ The majority of the publications capturing relevant events were case reports—these could not be incorporated in the quantitative meta-analysis for rates over time nor correlated with the volume of syringes being administered, potentially leading to underestimates.^6^ This is reflected in our findings: although 6 cases were documented for PureSense technology–based fillers from published case reports and series, and 11 events were reported for PNT (Preserved Network Technology)-based fillers during a PMS study, no events were observed from clinical follow-up for either technology—likely attributable to the small number of studies and short study duration.

Nevertheless, our study is the first to provide a comprehensive assessment by pooling various data points from available real-world evidence. Taken together, our study provides a descriptive landscape of the incidence and clinical characteristics of DIRs, adding to the existing body of literature that seeks to understand, prevent, and manage them effectively in practice. Future efforts may incorporate study designs, for example, large-scale longitudinal cohort studies, that enable the observational and systematic tracking of events over a longer period of time. Practitioners should be aware of product-specific factors that influence propensity for inflammatory responses, considering safety as a core and underlying tenet to achieve natural outcomes with HA fillers.^39^

## CONCLUSIONS

DIRs to HA fillers are rare but clinically significant events that can vary greatly in time to onset, presentations, severity, and duration of symptoms. Our study suggests that DIR risk may be influenced by product-specific factors rather than being uniform across HA fillers. Ultimately, these findings support informed risk–benefit discussions and reinforce the importance of understanding product characteristics to uphold patient safety in aesthetic practice.

## Supplementary Material

ojag165_Supplementary_Data
