# Supplementary material for: Incidence and Characteristics of Delayed Inflammatory Reactions Secondary to Hyaluronic Acid Filler Injections: A Systematic Review With Meta-Analysis
Source: Aesthet Surg J Open Forum. 2026 Sep 1;8:ojag165. doi: 10.1093/asjof/ojag165 (PMC13599015; doi:10.1093/asjof/ojag165)
Supplement: Supplementary file 1 [file ojag165_supplementary_data.zip › DIRs_Supplementary Table 1.docx]

**Supplementary Table 1. Search Strategy For Databases**

| Variable | Search Terms | Entries identified | |
| --- | --- | --- | --- |
|  |  | MEDLINE | Embase |
| Title and Abstract | (((hyaluron*) AND (filler* OR dermal filler* OR dermal implant* OR injection* OR facial OR cosmetic OR aesthetic)) AND (complication* OR safety OR adverse event* OR adverse effect*)) NOT "osteoarthritis" | 2,417 | 3,011 |
